# Geographical Variation in Breast Cancer Outcomes

**DOI:** 10.3390/ijerph14050523

**Published:** 2017-05-12

**Authors:** Peter Baade

**Affiliations:** Cancer Research Centre, Cancer Council Queensland, P.O. Box 201, Spring Hill, QLD 4004, Australia; peterbaade@cancerqld.org.au

Among females worldwide, breast cancer is the most frequently diagnosed cancer, accounting for 25% of all new diagnoses in 2012, and is the leading cause of cancer mortality (15% of total cancer deaths), particularly among less developed nations [1]. Incidence rates of breast cancer are generally higher in Northern America, Australia/New Zealand, and Northern and Western Europe, and lower in most African and Asian countries [2]. Factors associated with this international variation in incidence include those related to early detection, particularly the availability of mammography screening, as well as the prevalence of established risk factors, including overweight/obesity, use of menopausal hormone therapy, physical inactivity, and alcohol consumption [3].

There is, however, less international variation in breast cancer mortality rates, mainly because the countries with very high incidence also have high survival [1]. In many Western countries, mortality rates due to breast cancer are stable reducing, which has been attributed to the role of some combination of early detection using mammographic screening and improved treatment [3]. In contrast, mortality rates in many South American, African, and Asian countries are increasing [3].

While the international variation in incidence and mortality between countries is well documented, an increasing number of international studies [4,5,6] have consistently highlighted geographical inequalities within countries across the breast cancer continuum of care. Despite this, there is much still unknown. This Special Issue of the IJERPH titled “Geographical Variation in Breast Cancer Outcomes” provided an avenue for researchers to describe recent research studies designed to better understand the extent of these inequalities from a local, regional and international perspective, and, collectively, to increase our understanding of what factors are contributing to these inequalities.

Reflecting the wide scope and complexities of the breast cancer pathway, the twelve studies included in this Special Issue covered a variety of measures including screening [7], incidence [8], stage at diagnosis [9,10,11], quality of life [12], diagnostic interval [13], hormonal profile [14], surgical treatment [15], survival [9,10,16], and mortality [17,18].

Pleasingly, the included studies reported geographical patterns across a wide range of international environments, including Europe [7,12,17], North America [8,11,16], Asia [9,14,18], and the Oceania region [10,13,15].

If the diverse combination of measures and geographical scope limited our ability to report on common themes, this was made even more difficult by the differing methods of defining geographical variation across the studies. Types of comparisons included high income vs. middle to low income areas [7], public vs. private hospitals [9], measures of spatial clustering and variation [8,11,17], urban vs. rural areas [10,12,13], neighbourhood poverty [16], area disadvantage [13,15], housing type [14], variation between regions [18] and accessibility [15].

There are some disadvantages in having such a wide scope. For example, it was not possible to provide a consensus perspective of how the geographical patterns for key measures vary internationally. Direct comparisons across studies was difficult. However, rather than adding to the confusion, this heterogeneity of measures, geographies and approaches only served to confirm a remarkably consistent message that where women live has an impact on their experience and outcomes associated with breast cancer. Tellingly, those women who live in areas that are disadvantaged in some general way, whether economically, geographically or in regard to access to general services, also face poorer outcomes for breast cancer.

These results should provide motivation to act. First, to conduct more focussed research to better understand the reasons why these inequalities occur, and how they can be reduced. To this end, it would be advantageous to increase the use of similar methods and geographical entities across studies in different countries to facilitate direct comparisons; understanding why the extent of geographical variation differs between countries can provide important insights into the underlying causes of that variation. Second, to recognise that this knowledge forms only part of the equation. Unless knowledge converts into intervention by governments, policy makers, health professionals and other support personnel, then the type of research such as included in this Special Issue has served little purpose.

It is my hope that by providing novel insights into the geographical inequalities in breast cancer outcomes across the world, the twelve studies included in this Special Issue add to the weight of evidence that will motivate change to reduce these inequalities.
